# Fruit Quality Traits and Self‐ (In)compatibility Allele Status of Some Apricot ( *Prunus armeniaca* L.) Seedlings Obtained by Cross‐Breeding

**DOI:** 10.1002/fsn3.71196

**Published:** 2025-11-12

**Authors:** Derya Taşdemir Karaoğlan, Ercan Yıldız, Mehmet Yaman, Yazgan Tunç, Ali Khadivi

**Affiliations:** ^1^ Department of Horticulture Graduate School of Natural and Applied Sciences, Erciyes University Kayseri Türkiye; ^2^ Department of Horticulture, Faculty of Agriculture Erciyes University Kayseri Türkiye; ^3^ Republic of Türkiye, Ministry of Agriculture and Forestry, General Directorate of Agricultural Research and Policies, Hatay Olive Research Institute Directorate Hatay Hassa Türkiye; ^4^ Department of Horticultural Sciences Faculty of Agriculture and Natural Resources, Arak University Arak Iran

**Keywords:** fruit breeding, fruit quality, genetic, principal components analysis, self‐compatibility

## Abstract

Cross‐breeding in apricot ( *Prunus armeniaca* L.) is widely used to increase genetic diversity and develop new cultivars with desirable characteristics. In the present study, the morphological traits, chemical properties, and self‐compatibility status of 96 apricot seedlings from controlled hybridization and the two reference cultivars (Hacıhaliloğlu and Kabaaşı) were examined. The morphological traits' coefficient of variation (CV) ranged from 3.22% to 55.83%. Principal component analysis (PCA) revealed that the first three components accounted for 63.8% of the total variance. Traits important for table apricots, such as fruit weight, fruit width, fruit length, fruit height, and fruit flesh/pit ratio, showed a high contribution in PC1. While L*, b*, and chroma values showed the highest effect on PC2, dried apricot‐related characteristics such as soluble solids content (SSC), pH, fruit shape, fruit firmness, and seed weight showed the highest effect on PC3. As a result of the heatmap hierarchical clustering analysis, seedlings and reference cultivars were divided into two main groups with different subsets. In total, 76 out of 96 seedlings showed self‐compatible alleles. Seedlings 6, 3, 8, and 39, having high fruit weight, an important character in table apricots, and seedlings 34, 35, 47, and 68, showing high SSC, an important feature in dried apricots, were determined to be self‐compatible. Although seedling 3 was self‐incompatible, it attracted attention for its red fruit color and weight. The findings provide valuable information for apricot breeding programs. These findings will shed light on studies on developing new apricot varieties with self‐compatibility and high fruit quality.

## Graphical abstract

Hybrid genotypes 6, 3, 8, and 39 having high fruit weight, an important character in table apricots, and hybrid genotypes 34, 35, 47, and 68 having high SSC, an important feature in dried apricots, were determined to be self‐compatible. Although hybrid genotype 3 was self‐incompatible, it attracted attention with its red fruit color and weight. The findings obtained can be used in apricot breeding studies, and studies will be carried out on certifying prominent hybrids in the coming years.

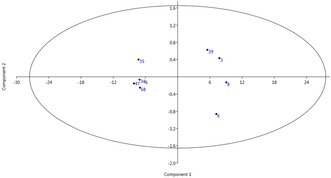

## 1 Introduction

Türkiye is a country where apricot ( *Prunus armeniaca* L.) cultivation is intensive globally and is the leader in production with 750,000 tons (FAO 2023). 43% of that production comes from Malatya province (TUİK 2024); almost all production is evaluated as dried apricots and their products. In addition to beta‐carotene and energy value, apricot fruit is rich in potassium and iron, which increase its nutritional value. In this respect, it is thought that interest in apricot fruit, which has the image of a “healthy fruit”, will continue (Gatti et al. 2009).

While breeding studies are being carried out to improve the quality and yield characteristics of apricot cultivars traditionally used, different breeding programs are being carried out, particularly considering consumer preferences. In these breeding programs, the aim is to develop new genotypes that are early and late maturing, adaptable to different ecological conditions, productive, have high physical (fruit size, firmness, etc.) and chemical quality (titratable acidity, taste, aroma, etc.), self‐ (in)compatibility, tolerant to diseases and pests, and adaptable to stress conditions (low temperature, drought, etc.) (Abbott et al. 2006; Reich et al. 2009; Tricon et al. 2009; Gatti et al. 2009; Zhebentyayeva et al. 2012; Yaman and Uzun 2020).

Apricot breeding studies in Türkiye began in the early 1939s. All local cultivars were developed by selection breeding (Asma et al. 2017). Later, to develop new cultivars, local and foreign cultivars were crossed with hybridization breeding studies, and the cultivars ‘Alata Yıldızı’, ‘Dr. Kaşka’, ‘Çağataybey’, ‘Çağrıbey’, ‘Şahinbey’, ‘Dilbay’ (Asma 2012), and ‘Eylül’ (Asma et al. 2018) were developed. Currently, in the Mediterranean Region, an important area for table apricot production, almost all of the early and mid‐early table apricot cultivars in commercial cultivation areas are of foreign origin. Breeding studies to develop early cultivars suitable for consumer preferences are very important in reducing this dependency. Local cultivars are entirely used in the production of dried apricots. However, although these cultivars have high % brix values, they are inadequate in some characteristics (self‐infertile, susceptible to diseases and pests, early flowering, etc.). In Türkiye, apricot breeding studies are ongoing on issues such as developing new drying cultivars that are tolerant to brown rot (*Monilinia* spp.) (Gülcan et al. 2006) and Sharka (Taşdemir Karoğlan et al. 2024) diseases, late flowering (Şahin et al. 2004), and have high fruit quality characteristics (Bilgin et al. 2016). Fruit size, fruit flesh firmness, color, taste, and aroma are the main criteria that constitute fruit quality in apricots (Khadivi‐Khub et al. 2013; Asma et al. 2017; Bircan et al. 2023; Ledbetter 2008; Yaman and Uzun 2021). New cultivars with superior characteristics to the cultivars on the market have not yet been developed. Some problems affect fruit set, quality, and yield in apricot. Apart from cultural practices, such as fertilization and irrigation, the most common problems are late spring frosts and partial or no yield due to self‐incompatibility. Therefore, a pollinator must be used to successfully pollinate self‐incompatible fruit species (Ortega and Dıcenta 2004). Self‐ and inter‐(in)compatibility in apricot has been studied in traditional cultivars from regions such as China, Hungary, Morocco, Spain, North America, Tunisia, and Turkey. Although some breeding programs in Spain, France, Italy, and the USA have evaluated new releases, the pollination requirements of many cultivars remain unknown (Herrera et al. 2018). Studies have shown that the incompatibility mechanism in apricots is gametophytic incompatibility controlled by a pair of S alleles (Yılmaz et al. 2016). This mechanism is regulated by a multiallelic S‐locus, which includes the pistil‐expressed S‐RNase and the pollen‐expressed F‐box (SFB) protein that inhibits pollen tube growth (Gordillo‐Romero et al. 2020). It is also known that S allele genes benefit yield and create genetic diversity in intra‐ and inter‐species breeding studies (Gordillo‐Romero et al. 2020; Yaman and Uzun 2020). Using different pollinators affects many characteristics, such as the amount of soluble solid content, size, and shape, which are known as quality traits in fruits (Güneş 2006).

In apricot, both traditional and molecular methods are used to determine the self‐incompatibility status of an accession. However, since the traditional breeding method is time‐consuming and affected by environmental conditions (Zhebentyayeva et al. 2012), molecular techniques have been used in determining self‐incompatibility in recent years (Burgos et al. 1998; Halász et al. 2005, 2007; Yılmaz et al. 2013). The self‐compatibility (SC) allele dominates over the self‐incompatibility (SI) alleles. The SRc‐R and SRc‐F markers associated with these SC alleles were developed by Romero et al. (2004) and Vilanova et al. (2005). It is stated that the band obtained with a size of approximately 353 base pairs using the developed primer pair appeared in apricot genotypes showing the self‐compatibility (SC) allele.

The present study examined fruit quality traits and self‐incompatibility status of some apricot seedlings obtained by cross‐breeding.

## 2 Materials and Methods

### 2.1 Plant Material

In the present study, 96 apricot seedlings located in the Battalgazi campus of Malatya province of Türkiye, and the most preferred cultivars in the market, Hacıhaliloğlu and Kabaaşı, were used as material. (Table 1).

**TABLE 1:** Apricot seedlings studied and their origin.

Seedlings	Original parents
1, 3, 30, 50, 51, 59, 82, 83	Kabaaşı × Roxana
2	Çataloğlu × Alyanak
4, 5, 6, 7, 8, 13, 14, 15, 16, 17, 18, 25, 26, 27, 28, 29, 41, 42, 49, 69, 73, 77, 81	Çataloğlu × Roxana
9, 10, 11, 12, 37, 58	Hacıhaliloğlu × Luizet
19, 20, 21, 36, 43, 44, 52, 53, 54, 74	Hacıhaliloğlu × Roxana
22, 24, 70, 76	Aprikoz × Kabaaşı
23	Aprikoz × Hacıhaliloğlu
94	Hacıhaliloğlu × Alyanak
31	Hacıhaliloğlu × Zard
32, 45	Kabaaşı × Zard
33, 34, 35, 38, 62, 78, 87, 88, 92	Kabaaşı × Hasanbey
39, 40, 61, 80	Hasanbey × Roxana
46, 47, 63, 64, 72, 79, 86	Kabaaşı × Paviot
48, 95	Adilcevaz × Özal
55, 75, 84	Kabaaşı × Marküleşti
56, 57	Hacıhaliloğlu × Marküleşti
60	Çataloğlu × Markuleşti
65, 89, 90	Hacıhaliloğlu × Paviot
66, 67, 91, 93	Kabaaşı × Gü 52
68	Çataloğlu × Gü 52
71	Kabaaşı × Luizet
85	Çataloğlu × Paviot
96	Hacıhaliloğlu × Güz Aprikozu
97, Reference cultivar	Kabaaşı
98, Reference cultivar	Hacıhaliloğlu

### 2.2 Morphological and Chemical Analyses

During the 2 years (2022–2023), fruits were harvested at the maturity stage. Morphological and chemical characteristics were examined as fruit width (mm), fruit length (mm), fruit height (mm), fruit shape, fruit weight (g), stone weight (g), fruit flesh/stone ratio, fruit flesh firmness (kg/cm^2^), soluble solid content (SSC, %°Brix), titratable acidity (TA, %), pH, fruit color (L*, a*, b*, Hue, and croma values).

### 2.3 Molecular Analysis

#### 2.3.1 Identification of S‐Alleles

*DNA extraction*: The leaf tissue was ground in a Muller under liquid nitrogen, and then DNA was extracted using the CTAB method according to the protocol of Doyle and Doyle (1990). DNA concentration was determined by spectrophotometric measurement. The DNA solution was brought to a concentration of 10 ng/μL and stored at −20°C.

*PCR amplification*: The method used to determine whether the genotypes considered are self‐compatible is a method using the primer combination SRc‐R (5′‐GGC CAT TGT TGC ACA AAT TG‐3′) and SRc‐F (5′‐CTC GCT TTC CTT GTT CTT GC‐3′) specifically for the relevant gene. The PCR concentration prepared for the primer pair used includes 75 mM Tris–HCl, pH = 8.8, 20 mM (NH4)2SO4, two mM MgCl2, 0.1% Tween 20, 100 μM dNTP mix, 0.2 μM of each primer, 1.0 unit of Taq DNA polymerase, and 30 ng DNA for a 25 μL amplification reaction. Temperature and cycling conditions were 3 min at 95°C. After the pre‐denaturation process, the samples were kept at 95°C for 30 s denaturation, 45 s at 54°C for primer annealing to DNA, and 1 min 15 s at 72°C for the extension phase. In addition, the samples were kept at 72°C for 10 min for the final extension phase (Vilanova et al. 2005). The obtained PCR products were run on a 2% agarose gel using 1xTBE buffer solution (89 mM Tris‐Cl, 89 mM boric acid, 20 mM EDTA), stained with ethidium bromide, and photographed under UV. Bandwidth was determined with a 100 bp ladder.

### 2.4 Data Analysis

To evaluate the morphological and chemical properties of the seedlings examined within the scope of the study, the minimum, maximum, and average values, standard deviation (SD), and the coefficient of variation (CV) showing the change between the data were calculated. Analyses based on morphological and chemical properties were performed using the JMP Pro 17.0 (SAS Institute Inc., Cary, NC, USA) statistical package program. Principal components analysis (PCA) was performed to determine the degree of influence of the examined traits and the relationship between genotypes. Heatmap hierarchical clustering analysis was performed to group the examined traits and genotypes.

## 3 Results and Discussion

Data on the morphological and chemical properties of the 96 seedlings and Hacıhaliloğlu and Kabaaşı cultivars are presented in Table 2. Variations were observed in terms of the examined traits. The coefficient of variation showing the change in properties was the highest in the titratable acid value (CV = 55.83%), followed by a* (CV = 51.39%) and fruit firmness (CV = 44.66%) values, and the lowest in fruit shape (CV = 3.22%). Traits with a CV value above 20% show more distinct differences among seedlings, and these traits can be used to separate these genotypes. It was found that the CV value was greater than 20% in four of the 16 analyzed traits (fruit weight, fruit firmness, a*, and titratable acidity). In a study on morphological traits in apricot, CV values were over 20% in 40 of 53 seedlings, and the highest CV value (95.68%) was observed in fruit relative area over color, while the lowest CV value was observed in fruit length/fruit ventral width (11.66%) (Rezaei et al. 2020).

**TABLE 2:** Fruit characteristics of apricot seedlings and reference cultivars.

No	Trait	Unit	Min	Max	Mean	SD	CV (%)
1	Fruit width	mm	25.09	42.97	35.67	3.53	9.90
2	Fruit height	mm	27.80	46.62	38.73	3.55	9.16
3	Fruit length	mm	30.35	50.65	39.64	4.23	10.67
4	Fruit shape	Code	1.00	1.19	1.09	0.03	3.22
5	Fruit weight	g	20.90	60.25	33.39	8.73	26.15
6	Stone weight	g	1.60	4.13	2.39	0.46	19.47
7	Fruit flesh/stone ratio	Code	7.67	18.50	13.00	2.37	18.21
8	Fruit firmness	kg/cm^2^	0.52	5.74	2.66	1.19	44.66
9	Soluble solid content	°Brix	8.70	28.00	19.03	3.54	18.61
10	Titratable acidity	%	0.14	1.61	0.60	0.33	55.83
11	pH	Code	3.02	5.10	3.89	0.50	12.81
12	L*	Code	32.09	79.46	63.10	7.85	12.44
13	a*	Code	−7.55	24.28	12.23	6.29	51.39
14	b*	Code	17.56	40.32	30.90	4.41	14.28
15	Hue	Code	43.69	102.60	68.29	11.39	16.67
16	Croma	Code	19.24	40.91	33.90	3.77	11.12

Abbreviations: CV, coefficient of variation; SD, standard deviation.

Among morphological traits, fruit width varied between 25.09 and 42.97 mm, fruit height varied between 27.80 and 42.97 mm, and fruit length varied between 30.35 and 50.65 mm. In addition, the highest CV value was 26.15% in fruit weight, while the lowest was 9.16% in fruit height. Studies conducted in different regions of Türkiye have detected differences in the size of apricot fruit (Gecer et al. 2020; Akca and Askin 1995; Asma and Ozturk 2005; Yılmaz et al. 2012). While fruit weight varied between 20.90 and 60.25 g in our study, it was found to be between 21.16 and 38.24 g in 8 accessions of the Aprikoz cultivar (Karataş 2021) and 21.16 and 38.24 g in Türkiye Malatya national apricot cultivars (Akin et al. 2008). Also, Milošević et al. (2014) found the fruit weight of 13 seedlings to be between 37.09 and 81.60 g. In this study, the fruit width was between 47.18 and 69.28 mm, and the fruit length was between 42.38 and 69.28 mm.

Fruit shape value 1 is round, while those greater than 1 are oval (Karataş 2021). Since the fruit shape in our study varied between 1 and 1.19, it can be said that most of the apricot seedlings tended to have an oval shape.

Stone weight varied between 1.60 and 4.3 g in seedlings and cultivars. Different researchers reported that stone weight values ranged from 2.98 to 5.01 g (Milošević and Milošević 2010), 6 g to 5.5 g (Rezaei et al. 2020), and 1.93 to 4.21 g (Yaman and Turan Sirke 2021). The fruit flesh/stone ratio CV value was 18.27%, and the average value of this character was 13. Karataş (2021) found the fruit flesh/stone ratio of eight accessions to be 8.67 (AP8) and 13.33 (AP4). Yılmaz et al. (2012) reported the flesh/stone ratio on Levent and Ozal apricot cultivars grown in Malatya between 7.9010.38 and 10.38 and 10.94 and 13.58, respectively, according to years.

In apricot fruit, firmness is an important quality criterion as it provides textural durability during transportation and marketing. The CV value of fruit firmness was 44.66%. The firmness value was between 0.52 and 5.74 kg/cm^2^. In a study conducted on early‐maturated 14 apricot cultivars grown in the Mediterranean region, the fruit firmness value was between 1.1 and 4.8 kg/cm^2^ (Caliskan et al. 2012), while in another study conducted on wild apricot genotypes, the fruit firmness values varied between 2.58 and 8.33 kg/cm^2^ (Karaat and Serce 2019).

Color values in apricot fruit provide critical information in terms of fruit quality, harvest time determination, and consumer preferences. Some variations in fruit color values were observed. The highest CV value was observed in the a* value (51.39%), representing the red color value. In comparison, the lowest CV value was observed in the croma value (11.12%), representing the color's liveliness and intensity. The average L*, a*, b*, Hue, and chroma values were determined as 63.10, 12.23, 30.90, 68.29, and 33.90, respectively. In a study, L value varied between 52.5 and 62.2, a* value ranged from 10.7 to 19.9, and the b* value ranged from 20.4 to 28.9 (Akin et al. 2008).

The most important chemical quality characters are soluble solid content (SSC), titratable acidity (TA), and pH. These characteristics directly affect the sensory properties, processing ability, shelf life, and consumer preference of the fruit. In terms of the specified characteristics, the highest CV values were in TA (55.83%), SSC (18.6°Brix), and pH (12.81%), respectively. In addition, a significant variation was observed among genotypes in terms of SSC value. According to Rakida (2023) research, the SSC value of apricot types ranged from 11° to 24°Brix. Yılmaz et al. (2013) reported that the SSC value of apricots cultivated in Türkiye ranged from 12.5° to 22.3°Brix. Ayanoğlu and Kaşka (1995) stated that the taste and flavor are perfect when the°Brixvalue is over 20 in apricot genotypes. It has also been emphasized that apricots above this value have high drying values. In our study, there are seedlings above this value, and thus, they have dried fruit characteristics. As apricot fruits ripen, the SSC value increases and the TA value decreases. At high TA values, the fruit tastes sour (Kader 2008). The TA varied between 0.14% and 1.61%. In a study conducted on 26 wild apricots in the Aras Valley, the TA was between 1.09% and 1.89% (Gecer et al. 2020), while in another study of Turkish apricot cultivars, TA varied between 0.80% and 1.00% (Akin et al. 2008). In a study, the TA was 2.40% to 1.93%, and the pH ranged from 3.01 to 3.25 in the Mogador and Mikado cultivars with earliness characteristics (Çalışkan et al. 2021).

### 3.1 Principal Components Analysis (PCA)

PCA is widely used to explain the degree of effect of the studied traits or variations between genotypes (Sümbül 2025). The first three principal components save significant time characterizing genotypes (Iezzoni and Pritts 1991; Yildiz et al. 2021; Yildiz et al. 2023). PCA analysis has been widely used in different fruit species, such as grape (Sümbül et al. 2023), berberis (Yaman et al. 2024), plum (Sümbül et al. 2024), and mulberry (Sümbül 2025), as well as in apricot collections (Badenes et al. 1998; Guerrieri et al. 2001; Azodanlou et al. 2003) to determine the genetic relationships between genotypes and to examine the correlation between fruit traits, tree traits, and phenological traits. The fact that the eigenvalues are greater than 1 in the PCA indicates that the central component weight values are quite reliable (Mohammadi and Prasanna 2003; Jeffers 1967).

**FIGURE 1:**
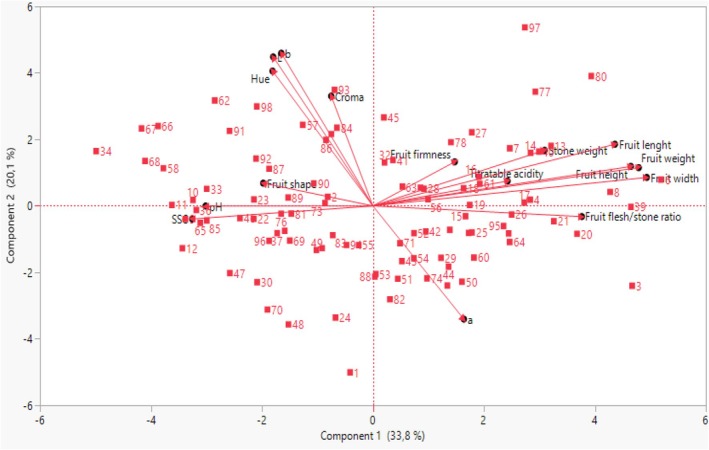
Distribution of apricot seedlings and cultivars according to the first and second principal components obtained from pomological data (PC1 *x*‐axis and PC2 *y*‐axis).

As a result of the principal component analysis (Table 3, Figure 1), there were five principal components with eigenvalues greater than 1. The eigenvalues of the PC1, PC2, PC3, PC4, and PC5 are 5.33, 2.79, 2.05, 1.32, and 1.20, respectively. While all of these components explained 79.37% of all features, the first three components explained 63.58% of all features. Mohammadi and Prasanna (2003) reported that the total variation of the first three components above 25% is important in showing heterogeneity in the gene pool. Our study showed that the variation level of the first three components (63.58%) is relatively high, approximately 2.5 times higher than the stated 25% rate. In the studies conducted on apricot, like our study, the cumulative percentage ratio of the first three main components was found to be above 25%, that is, 54%, 70%, and 73%, respectively (Reza et al. 2014; Asma and Ozturk 2005; Yılmaz et al. 2012). Each component has a different effect on pomological properties. The PC1 explained 33.30% of the properties, the PC2 explained 17.75%, and the PC3 explained 12.8%. In the study by Rakida (2023), PC1, PC2, and PC3 explained 11.11%, 7.39%, and 7.35% of the total variance, respectively (25.85% in total). In addition, in each PC, if the values of the components are 0.3 and above in terms of the examined criteria, they are considered to have significant importance (Brown 1991). According to Table 3, fruit width, fruit height, fruit length, fruit weight, and fruit flesh/stone ratio were placed in PC1; L, b, and croma values were placed in PC2; fruit shape, stone weight, SSC, and pH in PC3; fruit shape, a, and croma values in PC4; and titratable acidity and croma in PC5. The contribution to the principal components varied in each examined feature. The highest contribution to PC1 was provided by fruit width (16.33%), fruit weight (15.63%), fruit length (14.68%), fruit height (13.27%), and fruit flesh/seed ratio (9.15%), respectively. While color values L (30.41%), b (27.78%), and chroma (24.45%) had significant effects on PC2, and pH (17.31%), SSC (14.19%), fruit shape (11.24%), and stone weight (9.44%) had high contributions to PC3. According to Kumari et al. (2015), PC1's dominant traits, which account for 46.68% of the total variation, include the number of fruits per plant, fruit length, weight, seed length, fruit diameter, and yield per plant. In PC2, the traits with the greatest impact are seed weight and length; in PC3, the dominant characteristics are the number of fruits per plant, TSS, and seed weight. PCA is an important method in plant breeding as it can easily classify hybrid genotypes with desired pomological characteristics.

**TABLE 3:** Principal component analysis (PCA) and contribution ratio based on 16 pomological data of apricot seedlings.

Pomological trait	PC1	Contribution rate (%)	PC2	Contribution rate (%)	PC3	Contribution rate (%)	PC4	PC5
Fruit width	0.40	16.33	0.02	0.04	0.12	1.40	−0.02	0.07
Fruit height	0.38	14.68	0.06	0.34	0.24	5.99	0.08	0.03
Fruit length	0.36	13.27	0.14	1.97	0.20	4.02	−0.02	0.02
Fruit shape	−0.16	2.43	0.10	1.05	0.34	11.24	0.31	−0.12
Fruit weight	0.40	15.63	0.05	0.29	0.21	4.37	0.02	−0.04
Stone weight	0.26	6.84	0.13	1.68	0.31	9.44	0.24	0.06
Fruit flesh/stone ratio	0.30	9.15	−0.09	0.74	−0.04	0.13	−0.22	−0.10
Fruit firmness	0.13	1.61	0.20	4.03	−0.40	15.65	−0.15	−0.36
SSC	−0.26	6.80	−0.06	0.42	0.38	14.19	−0.01	−0.01
Titratable acidity	0.20	4.06	0.02	0.06	−0.27	7.41	−0.28	0.44
p.H	−0.23	5.49	−0.08	0.60	0.42	17.81	−0.26	0.05
L*	−0.11	1.30	0.53	27.78	0.01	0.01	−0.16	0.02
a*	0.09	0.87	−0.24	5.58	−0.24	5.73	0.63	−0.17
b*	−0.10	1.09	0.55	30.41	0.00	0.00	0.03	0.18
Hue	−0.05	0.23	−0.08	0.57	−0.11	1.18	0.27	0.76
Croma	−0.05	0.23	0.49	24.45	−0.12	1.43	0.34	−0.06
Eigenvalue	5.33		2.79		2.05		1.32	1.20
Percent	33.30		17.45		12.83		8.28	7.51
Cumulative percent	33.30		50.75		63.58		71.86	79.37

Figure 1 shows the two‐dimensional scatter plot, which shows the apricot seedlings and local cultivars' locations relative to one another. Seedlings, reference cultivars, and pomological traits were distributed according to four different sides of the plots. Genotypes closer to each other on the plot are similar in terms of the examined characteristics. The genotypes 1, 3, 80, 77, and 34, as well as the Kabaaşı cultivar (97), which are located away from the center, differed from the other seedlings. Muradoğlu et al. (2022) observed that Turkish and foreign apricot cultivars were divided into three groups in the plot according to pomological characteristics. Turkish cultivars were in the first two groups and characterized by fruit and seed characteristics, while pH, TSS, and color characteristics characterized foreign cultivars. Albayrak et al. (2022) divided the scatter plot into eight groups regarding fruit traits with 43 wild apricot and five standard apricot cultivars. Differences between study results may be due to differences in genotypes and characteristics examined.

### 3.2 Heatmap Hierarchical Clustering Analysis

**FIGURE 2:**
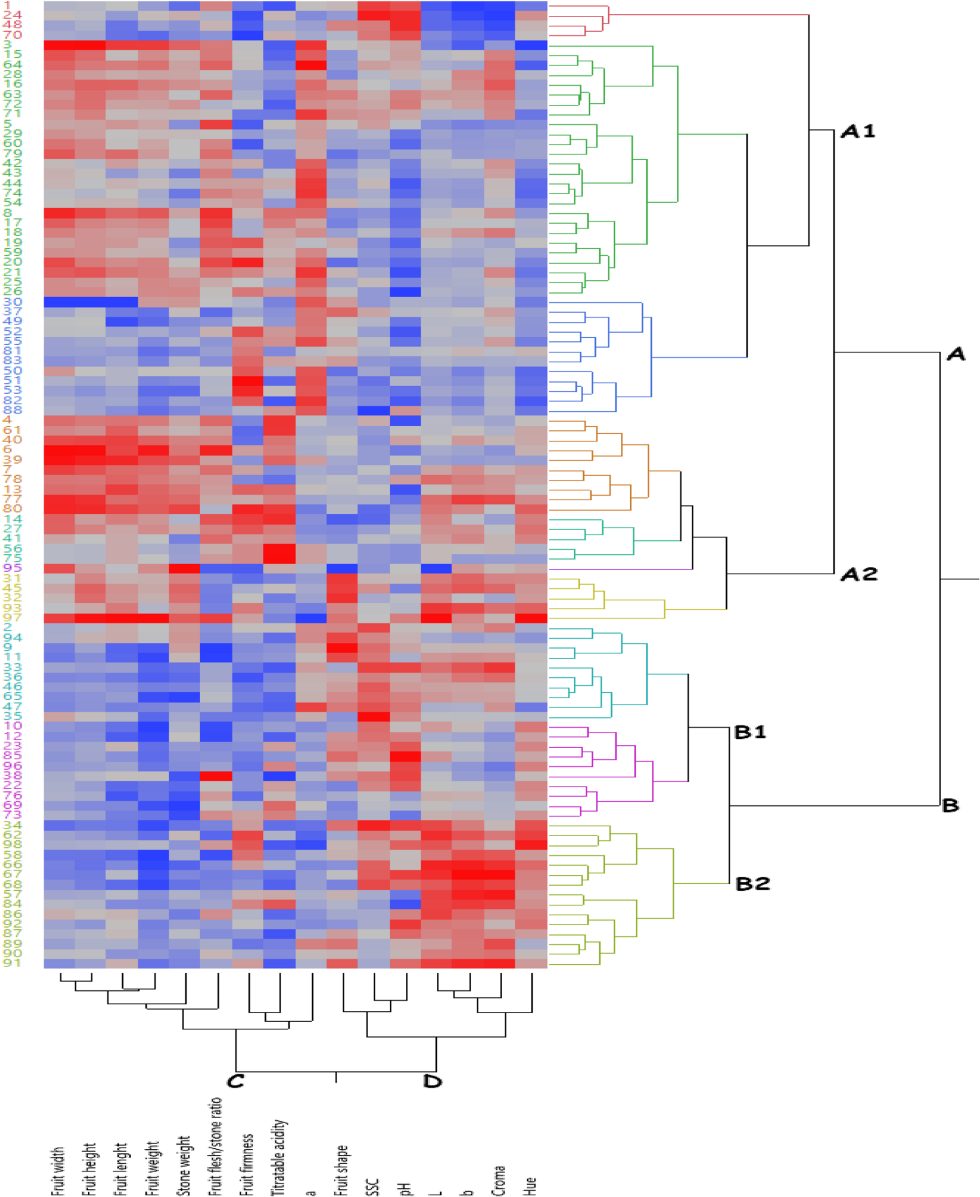
Heatmap hierarchical clustering analysis between apricot seedlings and reference cultivars and the pomological traits examined (red shifting colors on the heatmap scale indicate an increase, and blue shifting colors indicate a decrease).

Heatmap hierarchical clustering analysis was performed to classify the examined apricot individuals according to morphological and chemical characteristics. In the heatmap hierarchical clustering analysis, the change in color intensity from blue to red indicates the high trait values of the genotypes. In this study, as a result of cluster analysis, 96 seedlings were divided into two main clusters, including A and B (Figure 2). Group A consisted of a total of 62 seedlings and the Kabaaşı cultivar (97), while group B consisted of 34 genotypes and the Hacıhaliloğlu cultivar (98). There are 42 seedlings in the A1 group, while 20 genotypes and the Kabaaşı cultivar (97) are in the A2 group. There are 20 seedlings in the B1 group, while 14 seedlings and the Hacıhaliloğlu cultivar (98) are in the B2 group. The examined features are divided into two groups. Fruit width, fruit height, fruit length, fruit weight, stone weight, fruit flesh/stone ratio, fruit firmness, a*, and titratable acidity were in group C, and SSC, pH, L*, b*, Hue, croma, and fruit shape were in group D. According to the heatmap hierarchical clustering graph, the effects of the examined traits on the genotypes differed. As a result of these differences, the seedlings were clustered differently. In general, group A genotypes stand out in terms of the traits in group C. In contrast, group B seedlings stand out in terms of the characteristics in group D. Seedlings 1, 24, 48, and 70 in group A1 were grouped separately from other genotypes as they had high SSC and pH. Seedlings 95, the only one in the A2 group, stand out with its fruit width and stone weight. In addition, seedlings 31, 45, 32, and 93 in group A2 and Kabaaşı cultivar (97) exhibited high values in terms of color characteristics, unlike other genotypes in group A. Seedlings 91, 84, 57, 68, 67, and 66 in the B2 group exhibited higher L, b*, and chroma color traits than all other genotypes and cultivars. Heatmap hierarchical clustering analysis has been widely used to classify genotypes according to the studied traits in many fruit species (Yaman et al. 2023, 2024; Elikara et al. 2024; Say et al. 2024) as well as in apricot species (Basile et al. 2022; Mashhadi and Khadivi 2022; Rakida 2023).

### 3.3 Identification of Self‐Incompatibility Alleles

In the gel images obtained using the SRc‐R and SRc‐F primer pairs, the 353 bp band representing the SC allele was detected in 76 of the 96 apricot seedlings, whereas it was absent in 20 (Table 4). Herrera et al. (2022) reported the S‐genotypes of 66 apricot cultivars, identifying 49 as self‐compatible and 12 as self‐incompatible. Yılmaz et al. (2016) reported the presence of the SC allele in 23 genotypes among 236 apricot genotypes in the Malatya national apricot genetic resources parcel. Murathan et al. (2017) reported the presence of the SC allele in 57 of 127 F1 hybrid genotypes and 3 of 24 apricot genotypes. Pınar et al. (2015) reported the absence of the SC allele in all 43 apricot genotypes (34 wild apricot genotypes and 9 Turkish apricot cultivars). Similarly, Halász et al. (2013) found no SC allele in any of the 63 wild‐growing Turkish apricots. Oroji Salmasi et al. (2023) examined 25 apricot cultivars and identified genotypes 447 and 534 as late‐blooming and self‐compatible. Compared with these studies, the number of seedlings carrying the SC allele in our material is relatively higher. This difference may result from the specific combination of S‐alleles inherited from the parents and the genetic diversity among the seedlings.

**TABLE 4:** Self‐ (in)compatibility status of hybrid genotypes studied.

Genotype	SC alleles	Genotype	SC alleles	Genotype	SC alleles
1	SI	33	SI	65	SC
2	SI	34	SC	66	SC
3	SI	35	SC	67	SI
4	SC	36	SC	68	SC
5	SC	37	SI	69	SC
6	SC	38	SC	70	SI
7	SC	39	SC	71	SC
8	SC	40	SC	72	SC
9	SC	41	SC	73	SC
10	SC	42	SC	74	SC
11	SC	43	SC	75	SI
12	SC	44	SC	76	SC
13	SC	45	SC	77	SC
14	SC	46	SC	78	SC
15	SC	47	SC	79	SC
16	SC	48	SC	80	SI
17	SC	49	SC	81	SC
18	SC	50	SI	82	SC
19	SC	51	SC	83	SC
20	SC	52	SI	84	SC
21	SI	53	SC	85	SC
22	SI	54	SC	86	SI
23	SC	55	SC	87	SC
24	SI	56	SC	88	SC
25	SC	57	SI	89	SC
26	SC	58	SC	90	SC
27	SC	59	SC	91	SI
28	SC	60	SC	92	SC
29	SC	61	SC	93	SI
30	SC	62	SC	94	SC
31	SC	63	SC	95	SI
32	SC	64	SC	96	SI

Abbreviations: SC, self‐compatibility; SI, self‐incompatibility.

### 3.4 Combined Evaluation of Fruit Characteristics and Self‐Incompatibility Mechanisms

In apricot cultivation, the varieties are desired to be self‐compatible and superior in terms of fruit quality characteristics. When the heatmap hierarchical clustering analysis graph is examined, the Kabaaşı cultivar (97) in terms of fruit weight, seedlings 38, 6, 8, 5, 17, 20, and 19 in terms of fruit flesh/stone ratio, and genotypes 51, 20, 80, 53, 14, and 62 in terms of fruit firmness are the prominent hybrids among the important fruit quality traits in table apricots. Regarding SSC content, an important quality criterion for dried apricots, seedlings 35, 24, 34, 1, 33, 2, 68, 47, 33, and 46 were the prominent seedlings. Seedlings 35, 34, 68, and 46 were self‐compatible. The color values of the seedlings varied between yellow and red. The color of apricots varies according to consumer preferences. According to the high and low hue values expressing the red color, seedlings 3, 64, 8, 21, 55, 50, 51, 82, 88, and 47 were prominent among the seedlings. However, seedling 3 was determined to be self‐incompatible. Although the seedlings that stand out in fruit quality characteristics vary, the most prominent ones were determined to be self‐compatible.

Although the seedlings that stand out in fruit quality characteristics vary, the most prominent ones were determined to be self‐compatible. Unlike hybrid genotype 3, which was not self‐compatible, seedlings 6, 8, and 39, which were self‐compatible, stand out in terms of fruit weight and flesh/stone ratio, among the main objectives of apricot breeding.

## 4 Conclusions

This study examined apricot seedlings derived from controlled cross‐breeding by integrating fruit quality traits with self‐ (in)compatibility status. The present research combined morphological and S‐allele characterizations to identify promising genotypes for both fresh consumption and drying purposes. The finding that 76 out of 96 seedlings carried self‐compatible alleles is particularly valuable, as it highlights the feasibility of developing cultivars that combine consumer‐preferred fruit traits with stable pollination and yield potential.

The identification of self‐compatible seedlings with superior table apricot characteristics (e.g., high fruit weight, desirable flesh‐to‐stone ratio, and attractive color) and those suited for dried apricot production (e.g., high soluble solids content) provides concrete breeding material that can reduce reliance on foreign cultivars in the Mediterranean region. Moreover, the observation that some self‐incompatible but phenotypically attractive seedlings (such as seedling 3) exist underscores the importance of integrating molecular tools in breeding strategies to balance fruit quality with reproductive efficiency.

Overall, this study contributes to apricot breeding by demonstrating that cross‐breeding strategies can simultaneously enhance fruit quality and self‐compatibility. These findings not only support the selection of elite seedlings for cultivar development but also enrich the existing knowledge of how genetic diversity in apricot can be harnessed to address key challenges such as self‐incompatibility, consumer preferences, and postharvest quality.

## Author Contributions

**Derya Taşdemir Karaoğlan:** data curation (equal), investigation (equal), methodology (equal), visualization (equal), writing – original draft (equal). **Ercan Yıldız:** conceptualization (equal), data curation (equal), formal analysis (equal), funding acquisition (equal), investigation (equal), methodology (equal), writing – review and editing (equal). **Mehmet Yaman:** investigation (equal), methodology (equal), project administration (equal), supervision (equal), writing – review and editing (equal). **Yazgan Tunç:** data curation (equal), software (equal), validation (equal), writing – original draft (equal). **Ali Khadivi:** formal analysis (equal), methodology (equal), resources (equal), validation (equal), writing – review and editing (equal).

## Consent

The authors have nothing to report.

## Conflicts of Interest

The authors declare no conflicts of interest.

## Data Availability

The data that support the findings of this study are available from the co‐corresponding authors upon reasonable request.
